# Strain Dependent Electronic Structure and Band Offset Tuning at Heterointerfaces of ASnO_3_ (A=Ca, Sr, and Ba) and SrTiO_3_

**DOI:** 10.1038/srep41725

**Published:** 2017-02-14

**Authors:** John D. Baniecki, Takashi Yamazaki, Dan Ricinschi, Quentin Van Overmeere, Hiroyuki Aso, Yusuke Miyata, Hiroaki Yamada, Norifumi Fujimura, Ronald Maran, Toshihisa Anazawa, Nagarajan Valanoor, Yoshihiko Imanaka

**Affiliations:** 1 Fujitsu Laboratories, Atsugi, Kanagawa-ken, Japan; 2 Innovator and Inventor Development Platform, Tokyo Institute of Technology,4259-J3-21 Nagatsuta, Midori-ku, Yokohama 226–8502, Japan; 3 Institute of Mechanics, Materials and Civil Engineering, Université Catholique de Louvain, B-1348 Louvain-la-Neuve, Belgium; 4 Graduate School of Engineering, Osaka Prefecture University, 1-1 Gakuen-cho, Naka-ku, Sakai, Osaka 599-8531, Japan; 5 School of Materials Science and Engineering, University of New South Wales, Sydney, 2052, Australia

## Abstract

The valence band (VB) electronic structure and VB alignments at heterointerfaces of strained epitaxial stannate ASnO_3_ (A=Ca, Sr, and Ba) thin films are characterized using *in situ* X-ray and ultraviolet photoelectron spectroscopies, with band gaps evaluated using spectroscopic ellipsometry. Scanning transmission electron microscopy with geometric phase analysis is used to resolve strain at atomic resolution. The VB electronic structure is strain state dependent in a manner that correlated with a directional change in Sn-O bond lengths with strain. However, VB offsets are found not to vary significantly with strain, which resulted in ascribing most of the difference in band alignment, due to a change in the band gaps with strain, to the conduction band edge. Our results reveal significant strain tuning of conduction band offsets using epitaxial buffer layers, with strain-induced offset differences as large as 0.6 eV possible for SrSnO_3_. Such large conduction band offset tunability through elastic strain control may provide a pathway to minimize the loss of charge confinement in 2-dimensional electron gases and enhance the performance of photoelectrochemical stannate-based devices.

The alkaline earth stannates ASnO_3_ (A=Ba, Sr, and Ca) are rapidly emerging as important materials for a range of applications. The large room temperature mobility of La doped BaSnO_3_ (BLSO) single crystals ~320 cm^2^/Vs, coupled with optical transparency, make BLSO a candidate transparent conducting oxide (TCO) to replace Sn doped In_2_O_3_ (ITO)^1^. The large mobility of BLSO has also been utilized to fabricate all-perovskite field-effect transistors with an epitaxial BLSO channel layer resulting in large on-off ratios^2^. In addition to these applications, the alkaline earth stannates have been considered as photocatalysts^3^, where the high mobility may provide for more efficient separation of photogenerated electron-hole pairs that is necessary to drive multi-electron chemistry^4^. Furthermore, theoretical studies have predicted remarkable properties of stannate-based heterostructures including hybrid improper ferroelectricity achieved through engineering of the octahedral tilting modes in bicolor ASnO_3_/ASnO_3_ (A=Ca, Sr, Ba) superlattices^5^ as well as electrically controllable two dimensional electron gases (2DEGs) at interfaces between CaSnO_3_ or ZnSnO_3_ and KTaO_3_ or KNbO_3_^6^. The potential of stannate based oxide devices being evidently so great, it has been suggested that stannates have the potential to be as impactful to future electronic devices as silicon is in present day^7^.

Band gaps and band alignments at heterointerfaces are critical parameters for the design of future devices envisioned for stannate based materials. The bandgap determines, in part, the degree of light absorption which is important for applications that require transparency, such as TCOs that have a diverse range of function including use as low resistance electrical contacts for light emitting diodes and solar cells^8^, as well as for applications requiring efficient light absorption such as photovoltaics and photochemical devices for hydrogen and oxygen evolution^3^,^4^. Band gaps in perovskite stannates have been predicted to exhibit a large volumetric strain dependence with a decrease in cell volume of ~3% in SrSnO_3_ resulting in an increase in the fundamental band gap of ~0.35 eV^9^. Band alignments, on the other hand, affect the contact resistance, overpotentials and photochemical activity in heterostructured photochemical devices^10^ and the degree of carrier confinement of 2DEGs^11^. An important role on the properties of such heterostructures is played by the elastic strain, with the effect of epitaxial strain on the electronic structure and band alignments (offsets)^12^ as a major focus for this study.

To harness the predicted large sensitivity of the band gap of stannates on volumetric strain in a beneficial way, the effect of strain on the electronic structure, including the valence and conduction band alignments, needs to be thoroughly understood. Specifically, for certain applications such as photocatalysts for oxygen evolution, strain-mediated tuning of the valence band edge^13^ (as opposed to tuning of conduction band edge) could offer additional degrees of freedom for achieving the desired functionality.

Here, we reveal strain dependent electronic structure of epitaxial stannate thin films and conduction band offset tuning at heterointerfaces of ASnO_3_ (A=Ca, Sr, and Ba) and SrTiO_3_. Epitaxial CaSnO_3_ (CSO), SrSnO_3_ (SSO), Ba_0.97_La_0.03_SnO_3_ (BLSO) and Sr_0.98_La_0.02_TiO_3_ (La-STO) thin films were grown via pulsed laser epitaxy. The elastic strain state of the CSO and SSO thin films was controlled through epitaxial growth of CSO and SSO on 2 at% Nb doped SrTiO_3_(001) single crystal substrates (referred to as NSTO) and predominantly strain relaxed BLSO-buffered NSTO substrates. Bulk SrSnO_3_ (SSO) and CaSnO_3_ (CSO) have orthorhombic structures with lattice parameters of √2 × 4.037 Å, 2 × 4.033 Å and √2 × 4.033 Å and √2 × 4.017 Å, 2 × 3.953 Å and √2 × 3.912 Å, respectively^3^,^9^,^14^. The surfaces of the NSTO and BLSO/NSTO substrates present different lattice matching to the SSO and CSO films with the NSTO substrate having a lattice parameter (a_o_ = 3.905 Å^12^) smaller than the pseudo cubic lattice parameters of bulk CSO and SSO (resulting in compressively strained CSO and SSO films) while the BLSO-buffered NSTO substrate (referred to as BLSO/NSTO) has a larger lattice parameter (bulk cubic BaSnO_3_, a_o_ = 4.116 Å^9^) than the pseudo cubic bulk lattice parameters of CSO and SSO.

The valence band (VB) alignments at the heterointerfaces of the epitaxial stannate thin films were characterized using *in situ* X-ray^15^ and ultraviolet photoelectron spectroscopies (XPS and UPS) while band gaps were evaluated by spectroscopic ellipsometry (SE). In addition to the four structures (CSO or SSO)/NSTO and (CSO or SSO)/BLSO/NSTO used for varying the elastic strain, for the *in situ* photoemission measurements BLSO and La-STO layers were also grown on top of the CSO or SSO layers to elucidate if the band alignments were commutative (band alignment does not depend on the growth sequence) as well as line shape changes observed for the Ba 3d core levels.

## Results

### Structural properties

**Figure 1:**
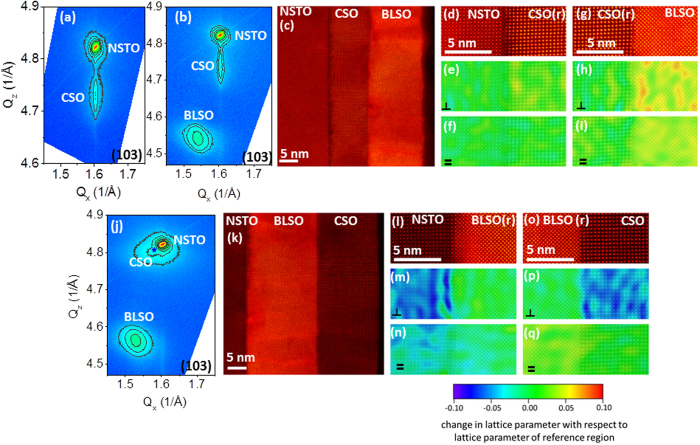
Structural characterization of CSO on NSTO and BLSO-buffered NSTO substrates. Reciprocal space maps of the (103) reflection for (**a**) CSO(12 nm)/NSTO, (**b**) BLSO(15 nm)/CSO(12 nm)/NSTO and (**j**) CSO(17 nm)/BLSO(20 nm)/NSTO heterostructures formed through step-wise growth during the *in situ* photoemission measurements. In (**j**) the position of the CSO maximum peak intensity is denoted by a blue star. HAADF STEM images of the structures for the RSM of (**b**,**j**) are shown in (**c**,**k**), respectively. (**d,g**) present enlarged HAADF STEM images at the interfaces between NSTO and CSO (**d**) and CSO and BLSO (**g**) for the BLSO/CSO/NSTO structure of (**c**) while (**l,o**) show the corresponding images for the CSO/BLSO/NSTO structure displayed in (**k**). (**e,f**,**h,i**) present GPA maps of the change in lattice parameter with respect to lattice parameter of the reference region (which is indicated by (**r**) and is always the first thin film layer grown on the NSTO substrate) along the growth direction (denoted by 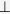) and an in-plane direction (denoted by=) in the vicinity of the NSTO|CSO and CSO|BLSO interfaces, respectively, for the BLSO/CSO/NSTO structure of (**c**). (**m,n**,**p,q**) show the corresponding GPA maps in the vicinity of the NSTO|BLSO and BLSO|CSO interfaces of the CSO/BLSO/NSTO structure of image (**k**). In order to highlight the relationship between the strain distribution and lattice structure, the HAADF STEM image expressed by a gray scale was overlaid onto each strain map shown by a rainbow color with a color bar scale given at the bottom of panels (**n,q**).

**Figure 2:**
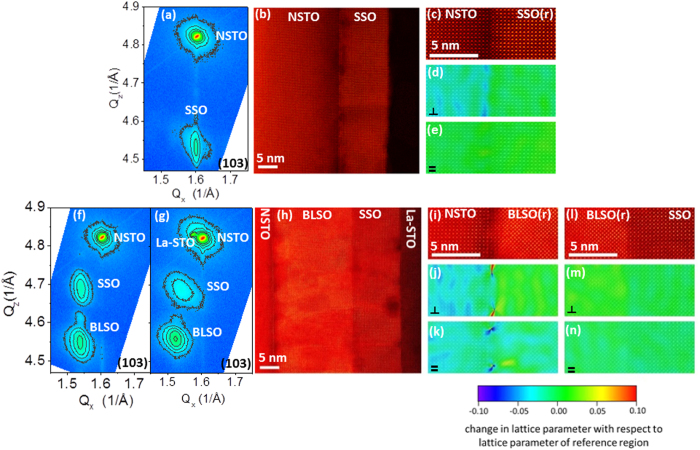
Structural characterization of SSO on NSTO and BLSO-buffered NSTO substrates. Reciprocal space maps of the (103) reflection for (**a**) SSO(11 nm)/NSTO, (**f**) SSO(11 nm)/BLSO(17 nm)/NSTO, and (**g**) La-STO(12 nm)/SSO(11 nm)/BLSO(17 nm)/NSTO heterostructures formed through step-wise growth during the *in situ* photoemission measurements. HAADF STEM images of the structures for the RSM data of (**a,g**) are shown in (**b,h**), respectively. (**c**) presents enlarged HAADF STEM images at the interface between NSTO and SSO of image (**b**), while (**i**,**l**) show the corresponding images at the interfaces between NSTO and BLSO and BLSO and SSO for the structure of image (**h**). (**d,e**) present GPA maps of the change in lattice parameter with respect to the lattice parameter of the reference region (which is indicated by (**r**) and is always the first thin film layer grown on the NSTO substrate) along the growth direction (denoted by 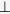) and an in-plane direction (denoted by=) for the SSO/NSTO structure in the vicinity of the interface of image (**b**), while (**j,k**,**m,n**) show the corresponding GPA maps in the vicinity of the NSTO/BLSO and BLSO/SSO interfaces of the structure of image (**h**). In order to highlight the relationship between the strain distribution and lattice structure, the HAADF STEM image expressed by a gray scale was overlaid onto each strain map shown by at rainbow color with a color bar scale given at the bottom of panels (**k,n**).

Figure 1 shows the reciprocal space maps (RSM) of the (103) reflection, high angle annular dark field scanning transmission electron microscopy (HAADF STEM) images, and geometric phase analysis (GPA)^16^ maps of the change in lattice parameter with respect to lattice parameter of the reference region along the growth and in-plane directions for BLSO(15 nm)/CSO(12 nm)/NSTO (Fig. 1(b–i)) and CSO(17 nm)/BLSO(20 nm)/NSTO (Fig. 1(j–q)) heterostructures formed through step-wise growth during the *in situ* photoemission measurements. Also shown in Fig. 1(a) is the RSM of a CSO(12 nm)/NSTO structure without the BLSO capping layer. Corresponding HAADF STEM images and RSM and GPA maps for SSO(11 nm)/NSTO and La-STO(12 nm)/SSO(11 nm)/BLSO(17 nm)/NSTO structures are presented in Fig. 2(a–e), respectively. Also shown in Fig. 2(f) is the RSM of a SSO(11 nm)/BLSO(17 nm)/NSTO structure without the La-STO capping layer. As indicated by the HAADF STEM images, the interfaces are abrupt, which was also confirmed with elemental chemical mapping across the CSO/NSTO interface of Fig. 1(d) using electron energy loss (EEL) spectroscopy (see Supplementary Fig. S1). As revealed in the RSM of Fig. 1(b,j), the 15 and 20 nm thick BLSO thin films are predominantly strain relaxed irrespective of whether they are synthesized directly on NSTO (BLSO in-plane and out-of-plane lattice parameters of a_BLSO_ = 4.11 ± 0.11 Å and c_BLSO_ = 4.13 ± 0.04 Å, respectively) or on top of the CSO layer (a_BLSO_ = 4.09 ± 0.11 Å and c_BLSO_ = 4.14 ± 0.05 Å). The BLSO films were largely strain relaxed at thicknesses as thin as ~2 nm, which is consistent with the small critical thickness reported for BLSO grown on STO^17^. As demonstrated in Fig. 1, the RSM (Fig. 1(a,b)) and GPA maps (Fig. 1(e,f)) show the CSO films grown on NSTO are coherently strained to the substrate through to the final thickness of the interface study with the relationship of lattice constants as deduced by GPA along the in-plane direction (*a*_NSTO _≈ *a*_CSO_ < *a*_BLSO_) and along the growth direction (*c*_NSTO_ < *c*_CSO _≪ *c*_BLSO_) consistent with those derived from RSM. In contrast, the CSO films grown on BLSO (Fig. 1(j–q)) are partially strain relaxed (*a*_NSTO_ < *a*_CSO_ < *a*_BLSO_ and *c*_NSTO _≈ *c*_CSO _≪ *c*_BLSO_).

For the SSO(11 nm)/NSTO structure shown in Fig. 2(b), the RSM and GPA maps of Fig. 2(a,e) reveal *a*_NSTO_ ≈ *a*_SSO_ for the in-plane lattice parameters. Thicker (78 nm) SSO films grown on NSTO were predominantly strain relaxed with a calculated^18^ unstrained film lattice parameter of 4.03(5) ± 0.05 Å which is close to that of bulk SSO and SSO thin films grown by molecular beam epitaxy (MBE)^18^. As demonstrated in Fig. 2(f), the SSO layer of the SSO(11 nm)/BLSO(17 nm)/NSTO structure without a top 12 nm La-STO layer has *a*_SSO _≈ *a*_BLSO_ for the in-plane lattice parameters while the RSM for the structure with the La-STO capping layer (Fig. 2(g)) exhibits a broader SSO (103) peak that is shifted slightly off the BLSO (103) peak (*a*_SSO_ = 4.07 ± 0.16 Å and *a*_BLSO_ = 4.12 ± 0.08 Å). At the NSTO|BLSO interface, misfit dislocations are visible in the GPA maps of Fig. 2(j,k).

The lattice parameters and cell volumes for the top thin film layer of the BLSO/NSTO, SSO/NSTO, CSO/NSTO, SSO/BLSO/NSTO, and CSO/BLSO/NSTO structures (all without BLSO or La-STO capping layers) used to determine the influence of strain on the CSO and SSO band gaps characterized by SE are summarized in Table 1. As shown in Table 1, significant changes in the CSO or SSO cell volumes are induced by the epitaxial strain (see Supplementary Table S1 for lattice parameters of all the layers for all the structures). GPA and line profiles of the atomic spacing in-plane and along the growth direction across the full thickness of the thin film layers of the fabricated heterostructures demonstrate the strain in the CSO and SSO layers is not confined to the interface but extends throughout the film volume (see Supplementary Figs S2–S5).

### Band gaps

**Figure 3:**
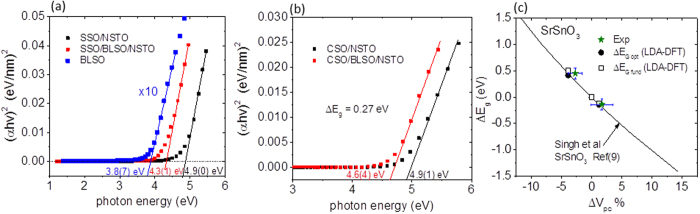
Optical absorption and strain dependence of the band gaps. (**a**) Optical absorption near the band edge in the form of a Tauc relation (α*hν*)^2^ versus the photon energy for a BLSO film grown on NSTO and SSO films grown on BLSO/NSTO and NSTO. In (**a**), the “x10” indicates (α*hν*)^2^ for BLSO has been scaled by a factor of 10. (**b**) presents data for CSO films grown on BLSO/NSTO and NSTO. (**c**) displays the bandgap change for the SSO data in (**a**) with respect to the percent volume change relative to the unstrained SSO pseudo cubic cell volume, denoted “Exp”, as well as that predicted using a function for SSO provided in ref (9) based on calculation results using DFT under the GGA and TB-mBJ potential functional. The error bars for ΔV_pc_ are derived from Table 1. Also shown are the changes in the optical ΔE_Gopt_ and fundamental gaps ΔE_Gfund_ with volumetric strain for SSO derived from DFT calculations under LDA used in the present study (see Supplementary Table S2).

As shown in Fig. 3, which presents the optical absorption near the band edge versus the photon energy *hν* in the form of a Tauc relation (α*hν)*^*2/n*^* *= *A(hν* − *E*_g_) where α is the absorption coefficient, *A* a constant, E_g_ the band gap, and n depends on the nature of the optical transition (n = 1 being directly allowed and n = 4 indirectly allowed^3^), the change in epitaxial strain resulted in significant changes in the band gaps of the SSO and CSO layers for the structures given in Table 1. Owing to the comparatively lower indirect band gap absorption expected in stannate thin films as discussed in ref. 18, only the direct band gap Tauc plots are presented in Fig. 3. The predominantly relaxed BLSO film with a Hall carrier density of 2.9 × 10^20^ cm^−3^ has a direct gap of 3.87 ± 0.1 eV, comparable to that of MBE grown BLSO films for similar carrier densities^18^.

As revealed in Fig. 3(a) for the compressively strained SSO film grown on NSTO (where the change in cell volume with respect to the unstrained pseudo cubic SSO cell volume, ΔV_pc,_ is −2.9%) a direct Tauc plot yields E_g_ = 4.90 ± 0.1 eV. On the other hand, the tensile strained SSO film grown on BLSO/NSTO with ΔV_pc_ = +1.7% has a direct gap of E_g_ = 4.31 ± 0.1 eV resulting in a significant band gap change of ΔE_g_ = 0.6 eV ± 0.2 eV and a band gap change per % unit cell volume change of −0.13 eV/%ΔV_pc._ Extrapolating ΔV_pc_ to zero yields a direct band gap of 4.4 eV for the SSO film which is close to that reported for predominantly unstrained SSO thin films grown by MBE^18^. For the compressively (coherently) strained CSO film grown on NSTO the direct band gap is 4.91 ± 0.1 eV which is reduced to 4.64 ± 0.1 eV resulting in a gap change per % unit cell volume change of −0.12 eV/%ΔV_pc_ which is close to the case of the SSO thin film.

The band gap change for the SSO films in Fig. 3(a) and the strain-induced changes in the optical and fundamental gaps for SSO as determined from DFT used in the present study (see Supplementary Table S2), as well as that predicted using a function for the volumetric strain dependence of the fundamental gap of SSO provided in ref. 9, are displayed in Fig. 3(c). As revealed by Fig. 3(c), close agreement is obtained between the experimentally-determined strain induced changes in the direct band gaps, and both theoretical approaches.

### Valence band electronic structure

**Figure 4:**
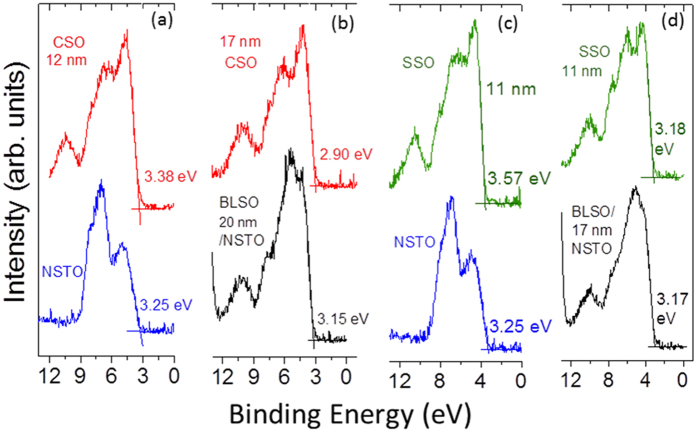
Strain dependent X-ray photoemission valence band spectra. XPS VB spectra acquired for (**a**) a 12 nm CSO film (final deposit) stepwise deposited on an NSTO substrate, (**b**) a 17 nm CSO film (final deposit) stepwise deposited on a BLSO-buffered NSTO substrate, (**c**) a 11 nm SSO film (final deposit) stepwise deposited on a NSTO substrate and (**d**) on a BLSO-buffered NSTO substrate. Note that the VB maximums of the 17–20 nm thick degenerately doped BLSO films are ~3.2 eV below the Fermi level while the fundamental gap of undoped BSO has been reported to be ~3 eV^9^. For this gap value, a Burstein-Moss shift expected for the measured BLSO carrier concentration would appear to suggest some degree of renormalization of the fundamental (indirect) gap^31^ has occurred.

Figure 4 presents the valence band (VB) spectra acquired for the NSTO and BLSO-buffered NSTO substrate and for the final thicknesses (given on the plot) of the CSO and SSO films after *in situ* stepwise growth. For both compressively strained CSO and SSO films grown on NSTO, the VB maximums of the CSO and SSO films are observed to lie towards higher binding energies (HBEs) by 0.48 eV and 0.39 eV, respectively, as compared to the respective VB maximums for the SSO and CSO films grown on BLSO/NSTO. Moreover, the line shapes of the CSO and SSO VBs are also strain-dependent, which is particularly notable for the SSO film in the region ~3 eV below the VB maximum (VBM). In this region, the second peak-like feature below the VBM observed for the tensile strained SSO film becomes markedly less pronounced for the compressively strained SSO film.

**Figure 5:**
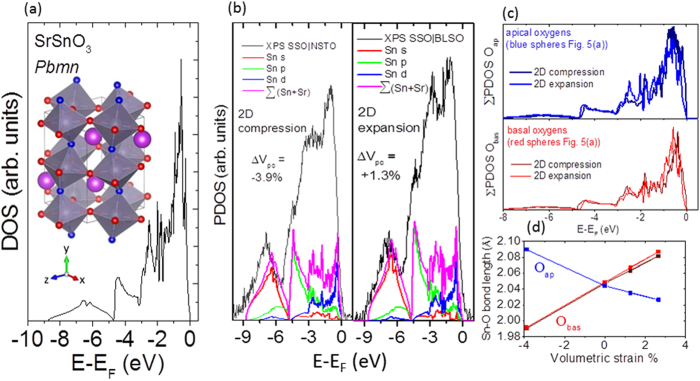
DOS of SrSnO_3_ with *Pbnm* orthorhombic symmetry and PDOS for various volumetric strain states of SrSnO_3_. **(a)** DOS of bulk orthorhombic SrSnO_3_ in *Pbmn* symmetry, (**b**) PDOS of orthorhombic SSO with different volumetric strain states achieved by compressing (left panel) or expanding (right panel) in-plane in 2D the supercell (see inset to (**a**)) to mimic the epitaxial strain. Also displayed in (**b**) are the XPS VB data of Fig. 4c (left panel) and 4d (right panel), respectively, that have been normalized to integrated intensity and referenced to the VB_max_ (equal to E_F_ for the PDOS data). (**c**) presents the PDOS summed over all apical (upper panel) and basal (lower panel) oxygens while (**d**) shows the change in Sn-O bond length for apical oxygens (blue curve) and basal plane oxygens (red curve) as a function of % volumetric strain. The crystallographic structure in (**a**) was visualized using VESTA^32^.

In contrast to the VB spectra of NSTO, which exhibits a two peaked structure consisting of a peak near the VBM due to predominately non-bonding O 2p states and a second peak ~2 eV lower in BE resulting from hybridized states of Ti 3d –O 2p orbital character^19^, the VB structure of the stannate thin films exhibits up to four well-resolved features. Such features can be correlated to features of the DFT-derived total density of states (DOS) and the partial density of states (PDOS) as demonstrated in Fig. 5(a–c), respectively.

The calculated DOS of bulk orthorhombic SrSnO_3_ (Fig. 5(a)) and the PDOS for different volumetric strain states of SSO, achieved by compressing or expanding in-plane (the XZ plane as shown in the inset to Fig. 5(a)) in two dimensions (2D) orthorhombic SrSnO_3_ unit cells to mimic the strained epitaxial films (Fig. 5(b,c)), reveals peaks near 6.5 eV, 4 eV, and 2.5 eV below VBM as well as a peak near to the VBM. Similar peaks are also observed in the DOS and PDOS of CSO (see Supplementary Fig. S6). Comparing the DOS and PDOS in Fig. 5 to the XPS VB data shown in Fig. 4 the peak ~6.5 eV below VBM and the shoulder ~4 eV below VBM, observed in the XPS VB spectra for all the stannate films, can be attributed to Sn 5s and Sn 5p states hybridized with O 2p states, respectively. The feature near 2.5 eV, which is markedly more pronounced in the case of the tensile strained SSO film grown on BLSO/NSTO, has hybridized character between predominately O 2p and Sn 5p and Sn 4d states. Similar to the experimental XPS VB data, the feature near 2.5 eV becomes less pronounced with increasing compressive volumetric strain, as demonstrated in Fig. 5(b). For both the calculated tensile (expanded in-plane and resulting in +1.3% volumetric strain) and compressive (matched in-plane to the relaxed 3.85 Å lattice parameter of STO and yielding −3.9% volumetric strain) strain states of SSO, the hybridized character of the PDOS changes with volumetric strain in a manner consistent with the XPS VB data (included in the plots for comparison). Both the PDOS summed over Sn and Sr (and weighted to account for differences in photoionization cross sections^20^) and the experimental XPS spectra loose relative spectral weight in the peak-like features near 2.5 eV below E_F_ and exhibit an increase in relative spectral weight near to the VBM with a change from tensile to compressive volumetric strain.

Furthermore, these PDOS features can be correlated to a change of Sn-O bond lengths, which depend on the direction of the bond with respect to the plane of expansion or contraction. The Sn-O bond lengths of apical oxygen atoms forming the oxygen octahedron surrounding each Sn atom (shown by the blue spheres in Fig. 5(a) and denoted by O_ap_) whose bond lengths have large projections normal to the XZ plane of compression (expansion), increase (decrease) with 2D compression (expansion). Conversely, the Sn-O bond lengths of the basal plane O atoms in the unit cell (shown by the red spheres in Fig. 5(a) and denoted by O_bas_), with large bond length projections in the plane of compression or expansion, decrease (increase) with compression (expansion). The changes of these bond lengths as a function of volumetric strain are shown in Fig. 5(d) and correspond to the Poisson effect on an atomic scale. As revealed by Fig. 5(c), which presents the PDOS summed over all O_ap_ atoms ∑PDOS O_ap_ (upper panel of Fig. 5(c)) and all O_bas_ atoms ∑PDOS O_bas_ (lower panel of Fig. 5(c)), in contrast to the O_bas_ atoms, the spectral weight for O_ap_ atoms near 2.5 eV markedly decreases with the 2D compression. Thus the compression-induced loss in spectral weight near 2.5 eV in the experimental VB spectra shown in Figs 4 and 5(b) is attributed to a decrease in Sn-O hybridization of apical oxygens hybridized with Sn p and d states owing to the 2D in-plane compression.

### Valence band offsets

**Figure 6:**
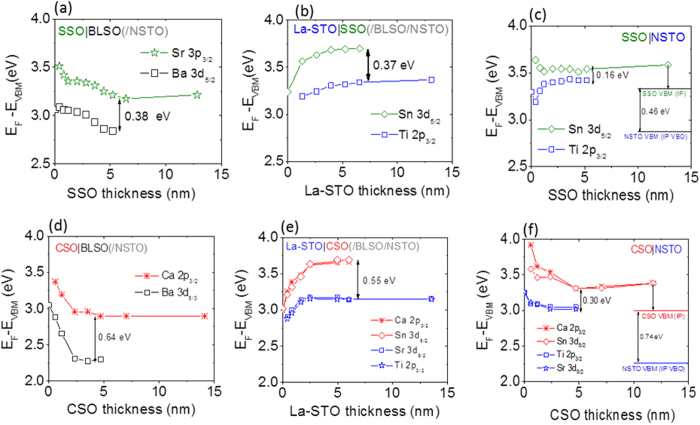
Thickness evolution of the core levels for step-wise growth interface formation. (**a**) Thickness evolution of the Sr 3p_3/2_ and Ba 3d_5/2_ core levels for stepwise growth of SSO on BLSO-buffered NSTO, (**b**) the Sn 3d_5/2_ and Ti 2p_3/2_ core level evolution for subsequent stepwise growth of an La-STO layer on SSO/BLSO/NSTO and (**c**) for stepwise deposited SSO on NSTO. (**d,e**) present comparable figures for stepwise growth of CSO on (**d**) BLSO/NSTO, (**e**) subsequent stepwise growth of an La-STO layer, and (**f**) on NSTO monitoring the thickness evolution of the Ca 2p_3/2_, Ba 3d_5/2_ and Ca 2p_3/2_, Ba 3d_5/2_, Sr 3d_5/2_, Ti 2p_3/2_ core levels, respectively.

The valence band offsets (VBOs) were determined using Kraut’s method by monitoring the thickness evolution of the core level (CL) spectra^19^,^21^ and for ease of comparison of VBOs, BE differences have been referenced to the respective thick film (final deposit) or substrate VB maximums of each layer forming the interface^19^,^21^. Figure 6 shows the thickness evolution of the core levels for chemical elements that are specific to each layer forming the interface.

For stepwise growth of tensile strained SSO on BLSO-buffered NSTO or subsequent growth of La-STO on SSO/BLSO/NSTO the CLs exhibit parallel shifts beyond ~1.2 nm and the VBOs are 0.4 ± 0.1 eV for both SSO|BLSO and La-STO|SSO with the SSO VBM lower in energy. The equivalent VBO of both interfaces suggests a small VBO at the La-STO|BLSO interface. Notably, the magnitude of the La-STO|SSO VBO is, within error, the same as that predicted by DFT where the VBO at the SSO|STO interface was 0.37 eV^22^. On the other hand as revealed in Fig. 6(c), for stepwise growth of the compressively strained SSO on NSTO the CL spectra exhibit a non-parallel, thickness-dependent energy separation between the VBM-referenced CL shifts. The Ti 2p_3/2_ CL emission has an initial shift to LBE and then a back bending shift of 0.25 eV towards HBE, while the Sn 3d_5/2_ exhibits a decrease towards lower binding energy (LBE) with the CL emission evolution approximately saturating after ~2 nm. For the thinnest deposit of ~0.4 nm the energy separation is 0.46 eV, similar to that for SSO deposited on BLSO, and decreases to 0.16 eV for deposited thicknesses greater than ~2 nm. The latter number for the SSO|NSTO interface is significantly smaller than that observed for the La-STO|SSO interface, with SSO stepwise deposited on BLSO-buffered NSTO, resulting in an apparent difference in the VBO depending on the stacking sequence.

A similar difference in the thickness evolution of the CL emissions is also observed between CSO stepwise deposited on BLSO-buffered NSTO and on NSTO as shown in Fig. 6(d–f). For CSO stepwise grown on BLSO-buffered NSTO the CLs exhibit parallel shifts after ~1.2 nm and the VBO at the CSO|BLSO interface is 0.6 ± 0.1 eV, with the CSO VBM lower in energy. On the other hand, for the compressively strained CSO stepwise deposited on NSTO the Ti 2p_3/2_ and Sr 3p_3/2_ CL emissions of the substrate have (within error) the same BE shifts while the Ca 2p_3/2_ and Sn 3d_5/2_ CL emissions show a significantly different evolution at thicknesses less than ~2 nm, indicating a difference in the evolution of the chemical bonding environment^23^ between Ca and Sn during the first 2 nm of CSO film growth. At the thinnest deposit of ~0.8 nm the energy separations between the CSO CLs and substrate Ti 2p_3/2_ and Sr 3d_5/2_ CLs (which overlap) varies from 0.5 to 0.8 eV for the Ca 2p_3/2_ and Sn 3d_5/2_ CLs, respectively. For the thickest deposit for which the substrate CLs are still observable, the Ca 2p_3/2_ and Sn 3d_5/2_ BE shifts coincide and the energy separation between the VBM referenced CL shifts is 0.3 ± 0.1 eV. The significantly smaller apparent VBO for the CSO|NSTO interface, as compared to that observed for the La-STO|CSO interface with CSO stepwise deposited on BLSO-buffered NSTO, also results in an apparent dependence of the VBO on the stacking sequence.

**Figure 7:**
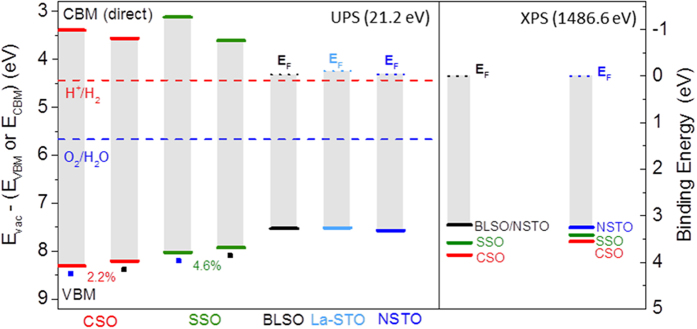
UPS valence band edges with respect to the vacuum level and XPS derived VBOs. Left panel: Valence band edges with respect to the vacuum level for the bare NSTO substrate and for the step-wise deposited CSO, SSO, BLSO, and La-STO thin film layers after the final deposit measured using *in situ* UPS with He I excitation (21.2 eV). Numbers on the plot for CSO and SSO indicate the % increase in cell volume from the compressively strained films deposited on NSTO (indicated by the small blue square) to the films deposited on BLSO-buffered NSTO (indicated by the small black squares) as determined from reciprocal space mapping (Table 1). The conduction band minimums (CBMs) for CSO and SSO are inferred from direct gap Tauc plots as shown in Fig. 3 (DFT electronic structure calculations for CSO and SSO indicate a small energy separation of ~0.1 eV between VB maximums for the direct and indirect optical transitions (see Supplementary Table 2)). Dotted lines for degenerately doped BLSO, La-STO, and NSTO represent the surface Fermi level position for the final deposit films (20 nm for BLSO and 15 nm for La-STO). The energetic position of the H^+^/H_2_ and O_2_/H_2_O standard redox potentials (pH 0) relative to vacuum (ref. 33) are shown for reference. Right Panel: Position of the VB maximums for the bare NSTO and BLSO-buffered NSTO substrates with respect to the Fermi level as measured using XPS. Also shown are the VB maximums of the SSO or CSO films grown on NSTO or BLSO-NSTO with energy separation between substrate and film VB maximums yielding the VBO as deduced from the thickness evolution of the CLs during stepwise growth of the films.

From the results described above, it may appear that the VB edges exhibit a relative shift in energy for the CSO or SSO films deposited on BLSO/NSTO or NSTO, which have different strain states that may affect the band alignment. To help ascertain if the VBM shifts in energy with strain, measurements of VB edges with respect to the vacuum level (the ionization potential IP for the insulting films) were performed after the final deposit of each layer (CSO, SSO, BLSO, and La-STO) during the step-wise heterostructure fabrication using *in situ* UPS with He I (21.2 eV) excitation. As demonstrated in the left hand panel of Fig. 7 and Table 2, the IPs reveal at most small strain-induced shifts of ~0.1 eV towards HBE for the VBM of CSO (and also for SSO) films grown on NSTO and BLSO/NSTO, indicating that the difference in apparent VBOs obtained from the stepwise XPS measurements are not caused by a strain-dependent energetic shift of the VB maximums.

As revealed in Table 2, for growth of CSO or SSO on BLSO/NSTO, the “ideal interface” VBOs (in the absence of interface states and dipoles) predicted by subtracting the vacuum-level-referenced VB edges of the materials forming the interface are, within measurement error, the same as the XPS derived VBOs. Moreover, the UPS measurements also reveal nearly the same energy difference from the vacuum level to the VB maximums of the BLSO, La-STO films and NSTO substrate, consistent with the equivalent VBOs of the La-STO|SSO and SSO|BLSO interfaces as deduced from XPS. However, these UPS-derived and XPS-derived VBOs differ markedly for the compressively strained CSO or SSO films grown on NSTO (whose XPS-measured values are significantly smaller). This is also demonstrated pictorially in Fig. 7 by comparing the difference in energy of the UPS measured VB edges between the thick films and respective substrates on which they were grown, and in the right hand side panel in Fig. 7, which shows the VBOs as derived from XPS measurements.

As the calculated values of VBOs based on measured values of the IPs as well as theoretical studies^24^ suggest the VB maximums do not exhibit large shifts in energy with strain over the experimentally accessed range, and also taking into account that the predicted UPS-derived ideal offsets are virtually the same as the measured XPS VB offsets for the films grown on BLSO/NSTO, we are led to consider other mechanisms for the difference in the magnitude of the VB offsets evaluated by XPS and UPS, for the heterostructures under compressive strain. Charge up might be suspected because, in addition to the VB spectra shown in Fig. 4, all CSO and SSO film core levels exhibited the same shift to HBE for the final deposit on NSTO relative those on BLSO-buffered NSTO. However, both substrate and film emissions would exhibit the same shift (for a uniform surface potential) towards HBE (in contrast to that observed in Fig. 6(c,f)) indicating that another mechanism should be at play here.

Alternatively, VBOs derived from photoemission measurements can also be influenced by photovoltages (PVs)^21^,^25^,^26^ resulting from the separation of photoexcited electron-hole pairs in the presence of built-in electric fields. The nonequilibrium carrier distribution results in a PV that opposes the built-in electric field, decreasing band bending. The back bending^26^ of the substrate Ti 2p_3/2_ CL evolution shown in Fig. 6(c) is indicative of a PV in the NSTO near the SSO|NSTO interface, decreasing the band bending in the NSTO interfacial depletion layer, thus resulting in a shift of the NSTO CLs to HBE. As shown in the XPS VB spectra of Fig. 4, the VBM of the 12 nm thick CSO film (final deposit) stepwise deposited on NSTO is shifted 0.48 eV towards HBE relative to the VBM of the final deposit CSO on BLSO/NSTO (Fig. 4). However the IP measurements reveal the CSO (on NSTO) VBM to lie only 0.1 eV towards HBE with respect to the vacuum level as compared to the VBM of the CSO film on BLSO/NSTO as shown in Fig. 7 and Table 2. This suggests the HBE shift of the CSO VBM on NSTO relative to that of CSO on BLSO/NSTO in the XPS spectra is due to a field-induced shift of VBM (PV in the presence of built-in fields), as the IPs are not affected by rigid shifts of the VB and vacuum level in each layer. The position of the CSO VBM corrected for this HBE shift is illustrated by the red line (denoted CSO VBM IP) in Fig. 6(f). Using the VBO for the “ideal interface” deduced from the UPS IP data for the CSO|NSTO interface results in a NSTO VBM lying 0.74 eV towards LBE from the CSO VBM IP energy level (blue line in Fig. 6(f) and denoted NSTO VBM (IP VBO)). As seen by comparing Fig. 6(f,d), the CSO VBM IP and NSTO VBM (IP VBO) energy levels for CSO stepwise deposited on NSTO are close to those measured for the CSO VBM and NSTO VBM for CSO stepwise deposited on BLSO-buffed NSTO, respectively. This suggests that, taking into account differences in built-in fields, the CSO and NSTO VBM positions are those represented by the red and blue lines, respectively, in the right part of Fig. 6(f), with the shift of these levels towards HBE being influenced by a PV. Similarly, correcting the VBM position owing to the difference in the XPS and IP values for SSO stepwise deposited on NSTO and BLSO-buffered NSTO leads to a SSO VBM shifted towards LBE as shown by the green line in Fig. 6(c) (denoted SSO VBM IP), while the NSTO VBM (represented by the blue line and denoted NSTO VBM IP VBO) is shifted 0.46 eV towards LBE from the SSO VBM IP level. This again supports the finding that the VBOs for the stannate films stepwise deposited on NSTO are those obtained after correcting for the influence of the PV. With these corrections, we can now infer that the VB maximums of the SSO and CSO films do not exhibit large shifts in energy with strain over the experimentally accessed range, irrespective on evaluating the VB offsets by UPS or XPS. Hence, any strain dependent band alignment would imply strain-tuning of the conduction band offset rather than that of the valence band offset.

The apparent decrease in the measured VBO for the CSO or SSO structures on NSTO can be understood by considering the effect of a PV on an interfacial n-type depletion layer in the NSTO and field effects in the CSO or SSO layers (see Supplementary Fig. S7). Under a photon flux, the separation of electron-hole pairs in the NSTO depletion layer decreases the band bending, causing substrate VB and core levels to shift towards HBE relative to the equilibrium Fermi level. In the CSO or SSO film, an internal field, supported by line shape broadening (see Supplementary Fig. S8), results in an upward sloped potential gradient (from substrate to film surface) partially compensating the HBE shift of the CSO or SSO CLs at the interface with increasing depth into the film, resulting in an apparent decrease in the measured VBO determined by monitoring the thickness evolution of the CLs.

The absence of a pronounced PV effect for the CSO and SSO films grown on BLSO-buffered NSTO substrates could be ascribed to the different depletion layer thickness expected between BLSO and NSTO, given the significant differences in the relative room temperature lattice permittivities of NSTO (~300) and BLSO (~25). This would result in a reduction of the depletion width by about a factor of ~4 for the same built-in potential and thus, for sufficiently thin depletion layers, the PV cannot be maintained across the layer. For a built-in potential of 0.8 eV (similar to the PV induced HBE shifts) the depletion widths are estimated as 89 Å and 22 Å for the NSTO and BLSO, respectively. In addition, the decrease in band gaps for the CSO or SSO films grown on BLSO/NSTO will also result in smaller conduction band offsets allowing more facile charge transfer across the film-substrate interfaces decreasing diode resistance.

## Discussion

Our results show that, in contrast to the case of the band gap where volumetric strain plays a dominant role^9^,^24^, the valence band electronic structure of CSO and SSO is dependent on directional bonding in a manner that correlated to a difference in the change of bond lengths between Sn and O atoms at apical and basal plane positions in the unit cell which is a consequence of the two dimensional compression or tension. Our results also reveal, however, that the valence band maximums exhibit at most small shifts of approximately 0.1 V over the volumetric strain range of −2.9% to +1.7%, rresulting in having most of the difference in band alignment, owing to a change in the band gaps with strain, at the conduction band edge. This could have important implications for future devices incorporating stannate oxide materials, as explained below.

Due to the small dependence of the valence band alignments on strain, the interfaces of our heterostructures are nearly commutative with respect to the VBOs (i.e., they don’t depend on the growth sequence). However, our study shows that the conduction band offsets are not commutative with changes in the growth order, thus resulting in a possibility to strain-tune them using epitaxial buffer layers. Indeed, conduction band offset differences as large as 0.6 eV have been demonstrated here as achievable for SrSnO_3_ deposited on NSTO and on BLSO-buffered NSTO.

Such large conduction band offset tunability through elastic strain controlled via buffer layers provides a pathway to enhance stannate device performance. For instance, this could be helpful for minimizing the loss of charge confinement in 2-dimensional electron gases, or enhancing the activity of photochemical devices for hydrogen evolution (the latter depending in part on the energetic separation of the conduction band minimum to the H^+^/H_2_ redox potential^3^). Furthermore, in addition to the possibility to use strain engineering in fabricating stannate based devices with new functionalities, the significant change in conduction band offsets with strain also implies that paying particular attention to strain management will be critical for avoiding adverse effects that might impact on device performance.

## Methods

Epitaxial thin films were grown by pulsed laser epitaxy (PLE) using a q switched Nd:YAG laser with a pulse rate of 10 Hz on 2 at% Nb doped SrTiO_3_ (001) substrates (NSTO). The substrates were first *ex situ* annealed at 1000 °C in a flowing 80% N_2_/20% O_2_ atmosphere for 3 h yielding a step-terrace surface with mixed SrO/TiO_2_ termination^27^. The NSTO substrates were then subjected to an *in situ* heat treatment prior to film growth or XPS/UPS characterization at 650 °C for 30 min in 100 mTorr oxygen and then cooled in vacuum to remove surface contaminations as verified by XPS.

Stannate thin films were deposited using CaSnO_3_ (CSO), SrSnO_3_ (SSO) and Ba_0.95_La_0.05_Sn_1.1_O_3_ (BLSO) polycrystalline ceramic targets while for La doped SrTiO_3_ thin films (La-STO) a single crystal Sr_0.97_La_0.03_TiO_3_ target was used for PLE. Growth conditions were carefully optimized to minimize mosaic spread and cation non-stoichiometry. All films were grown at 923 K. BLSO films grown at 0.1 mTorr O_2_ and 1.0 J/cm^2^, SSO films grown at 3.7 mTorr and 0.9 J/cm^2^, and CSO films grown at 0.3 mTorr and 0.7 J/cm^2^ were stoichiometric within resolution limits of inductively coupled plasma-optical emission spectrometry. Hall measurements of the BLSO thin films deposited on undoped SrTiO_3_(001) and LSAT(001) substrates revealed room temperature electron mobilities of ~50–60 cm^2^/Vs (~5 cm^2^/Vs for La-STO) and Hall carrier densities of ~2.9 × 10^20^/cm^3^ (3.4 × 10^20^/cm^3^ for La-STO) for 50 nm thick films.

Structural characterization by x-ray diffraction was carried out using a *Philips X’Pert Pro* thin film diffractometer. The errors in the in-plane and out-of-plane lattice parameters were estimated from the full width half maximums of the RSM (103) peak along the < 100 > and < 001 > directions of k-space, respectively (ref. 28). In order to reveal the detailed layered structure together with induced strain at atomic resolution, GPA and HAADF STEM was performed using a JEM-2100F TEM/STEM (JEOL, Japan), which was operated at 200 keV and equipped with a Cs-corrector (CEOS, Germany). The semi-angle of incident convergent electron beam and the range of annular dark-field detector were set to be 20 mrad and 70—180 mrad, respectively.

*In situ* XPS and UPS was performed using an ultra-high vacuum system combining a photoelectron spectrometer via a transfer chamber with a pulsed laser deposition chamber as described elsewhere^13^,^15^. Such *in situ* characterization allows the study of pristine surfaces and interfaces revealing electronic structure without the parasitic influence of adsorbates. All core levels presented in Fig. 6 exhibited thickness independent line shapes except for the Ba 3d core level for stepwise growth of CSO on BLSO/NSTO where for CSO deposits thicker than ~2.5 nm the Ba 3d core level line shape was asymmetric and was synthetically resolved into two SOS doublets with FWHM of 1.4 eV and separated by 1.7 eV (see Supplementary Fig. S9).

Band gaps were determined by modeling tan(Ψ), cos(Δ) data obtained from spectroscopic ellipsometry (SE) over the wavelength range of 230–850 nm using a SOPRA GES-5E spectroscopic ellipsometer and from 190–900 nm (280–900 nm with retarder) using a Sentech SE850 with rotating polarizer and analyzer and a retarder. A deuterium lamp was utilized for the ultra-violet range (UV) range and a halogen lamp for the visible-near infrared range. Dielectric functions were obtained for the NSTO substrates by direct inversion of the ellipsometric parameters and for single layer BLSO, CSO, and SSO films on NSTO and the top layer of CSO/BLSO or SSO/BLSO bi-layer films on NSTO by regression on the tan(Ψ), cos(Δ) data.

The first-principles calculations were carried out using the density functional theory (DFT) under the local density approximation (LDA), as implemented in the ABINIT package^29^. For all chemical elements, projected-augmented wave pseudo-potentials were used with plane-waves of electronic wave functions truncated at about 400 eV. Both valence and semi core electronic configurations, including specifically the Sn 4d, 5s and 5p electrons, the Ca 3s, 3p and 4s electrons, the Sr 4s, 4p and 5s electrons, and the O 2s and 2p electrons were considered into the calculations. The calculations were performed on 4 × 4 × 3 Monkhorst–Pack k-point meshes for geometry relaxations (that reduced the inter-atomic forces to less than 2 meV/Å) and on denser 12 × 12 × 12 k-point meshes for evaluating the densities of states (DOS).

Stannate structures had *Pbnm* orthorhombic symmetry or *I4/mcm* tetragonal symmetry^30^ in 20 atom unit cells and were either fully relaxed to provide bulk-like lattice constants and electronic properties or had the short axes compressed or elongated according to the strain imparted on the films by the substrates onto which they were grown. This model is in reasonable agreement with the RSM and GPA structural data that demonstrates the strain in the CSO and SSO layers is not confined to the interface but extends throughout the volume of the film (see Supplementary Figs S2–S5).The DFT-derived total energies of *Pbnm* or *I4/mcm* unit cells generated using the experimental lattice constants reported in Table 1 revealed the bulk-like orthorhombic symmetry is energetically favored for all the experimental structures: the DFT-derived energy of the *Pbnm* phase is 0.01 ÷ 0.4 eV and 1.27 ÷ 1.48 eV lower than the corresponding energy of the *I4/mcm* phase of same lattice constants, for SSO and CSO, respectively.

## Additional Information

**How to cite this article:** Baniecki, J. D. *et al*. Strain Dependent Electronic Structure and Band Offset Tuning at Heterointerfaces of ASnO_3_ (A=Ca, Sr, and Ba) and SrTiO_3_. *Sci. Rep.* **7**, 41725; doi: 10.1038/srep41725 (2017).

**Publisher's note:** Springer Nature remains neutral with regard to jurisdictional claims in published maps and institutional affiliations.

## Supplementary Material

Supplementary Information

## Figures and Tables

**Table 1:** Lattice parameters and cell volumes for BLSO, SSO, and CSO films with various strain states.

Structure (on NSTO)	In-plane lattice parameter a (Å)	Out-of-plane lattice parameter c (Å)	Volume (Å^3^)	ΔV_pc_ %	E_g_ (dir) ± 0.1 (eV)
BLSO	4.112 ± 0.08	4.129 ± 0.04	69.82 ± 2.6	0.1	3.87
SSO	3.919 ± 0.05	4.136 ± 0.02	63.75 ± 1.2	−2.9	4.90
SSO/BLSO	4.075 ± 0.08	4.021 ± 0.02	66.77 ± 1.9	1.7	4.31
CSO	3.905 ± 0.02	3.981 ± 0.05	60.71 ± 0.9	−2.3	4.91
CSO/BLSO	3.979 ± 0.14	3.922 ± 0.06	62.10 ± 3.2	−0.1	4.64

In-plane (denoted “a”) and out-of-plane (denoted “c”) lattice parameters, cell volumes, percent volume change with respect to pseudo cubic bulk ΔV_pc_ % (pseudo cubic bulk volumes: BSO = 69.73 Å^3^ ref. (1), SSO = 65.65 Å^3^ ref. (3), CSO = 62.12 Å^3^ ref. (3)), and band gaps deduced from a direct gap Tauc plot (Fig. 3) for BLSO, SSO, and CSO films with various strain states. For all structures the data is for the top thin film layer. The errors in a and c were estimated from the full width half maximums of the RSM (103) peak along the < 100 > and < 001 > directions of k-space, respectively. Regarding the bulk reference volumes as absolute, the error in ΔV_pc_ is equivalent to the error in the cell volume.

**Table 2:** UPS and XPS derived valence band offsets.

Structure	E_vac_ − E_VBM_ ± 0.05 (eV)	UPS VBO ± 0.1 (eV)	XPS VBO ± 0.1 (eV)
NSTO	7.566	—	
BLSO|NSTO	7.526	<0.1	<0.1
SSO|NSTO	8.024	+0.46	+0.16
SSO|BLSO(/NSTO)	7.923	+0.40	+0.38
La-STO|SSO(/BLSO/NSTO)	7.511	−0.41	−0.37
CSO|NSTO	8.305	+0.74	+0.30
CSO|BLSO(/NSTO)	8.207	+0.68	+0.64
La-STO|CSO(/BLSO/NSTO)	7.533	−0.67	−0.55

Vacuum level to VBM energy separations (E_vac_ − E_VBM_) of the top film surface of the given heterostructures as measured by UPS He I excitation, VBOs predicted by subtracting the vacuum level to VBM energy separations of the second column for the respective materials forming the interface (denoted UPS VBO), and XPS derived VBO for the given interfaces derived by monitoring the thickness evolution of the CLs emissions shown in Fig. 6. The adopted convention is the VBO of interface A|B is positive if the VBM of material A is lower in energy than the VBM of material B.
