# Supplementary material for: Strain Dependent Electronic Structure and Band Offset Tuning at Heterointerfaces of ASnO3 (A=Ca, Sr, and Ba) and SrTiO3
Source: Sci Rep. 2017 Feb 14;7:41725. doi: 10.1038/srep41725 (PMC5307316; doi:10.1038/srep41725)
Supplement: Supplementary Information [file srep41725-s1.pdf]

Strain Dependent Electronic Structure and Band Offset Tuning at Heterointerfaces of $ASnO_3$ (A = Ca, Sr, and Ba) and $SrTiO_3$

John D. Baniecki*[1], Takashi Yamazaki[1], Dan Ricinschi[2], Quentin Van Overmeere[3], Hiroyuki Aso[1], Yusuke Miyata[4], Hiroaki Yamada[4], Norifumi Fujimura[4], Ronald Maran[5], Toshihisa Anazawa[1], Nagarajan Valanoor[5], and Yoshihiko Imanaka[1]

[1]*Fujitsu Laboratories, Atsugi, Kanagawa-ken, Japan*

[2]*Innovator and Inventor Development Platform, Tokyo Institute of Technology,4259-J3-21 Nagatsuta, Midori-ku, Yokohama 226–8502, Japan*

[3]*Institute of Mechanics, Materials and Civil Engineering, Université Catholique de Louvain, B-1348 Louvain-la-Neuve, Belgium*

[4]*Graduate School of Engineering, Osaka Prefecture University, 1-1 Gakuen-cho, Naka-ku, Sakai, Osaka 599-8531, Japan*

[5]*School of Materials Science and Engineering, University of New South Wales, Sydney, 2052, Australia*

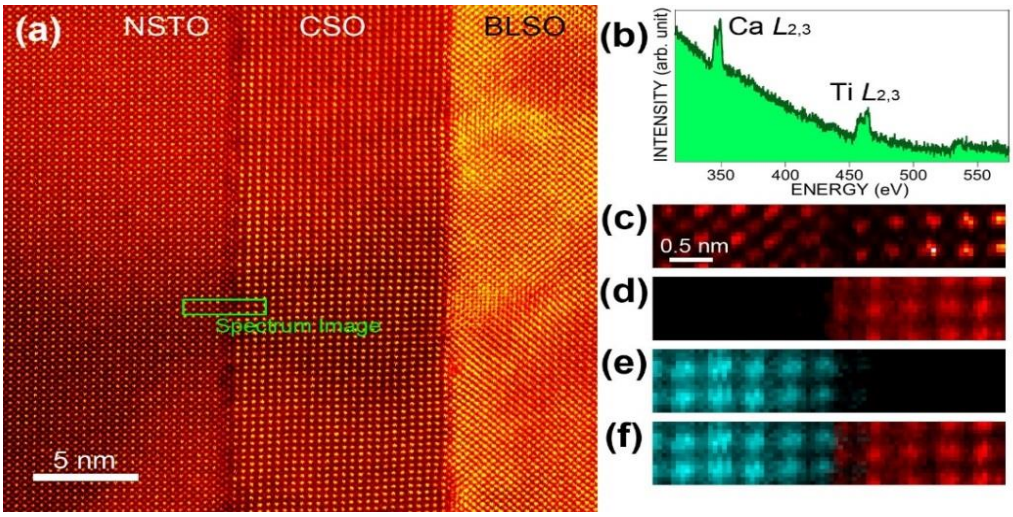

Supplementary Fig. S1. High angle annular dark field scanning transmission electron microscopy image of a BLSO/CSO/NSTO heterostructure. (a) High angle annular dark field scanning transmission electron microscopy (HAADF STEM) image of BLSO/CSO/NSTO. Fringed frame in green shows the range that has acquired the spectrum image. (b) the sum EEL spectrum of observed area, (c) simultaneously observed HAADF STEM image, (d) elemental chemical map of Ca $L_{2,3}$ edge (red), (e) elemental chemical map of Ti $L_{2,3}$ edge (cyan), and (f) overlay image of elemental chemical maps of Ca $L_{2,3}$ edge and Ti $L_{2,3}$ edge.

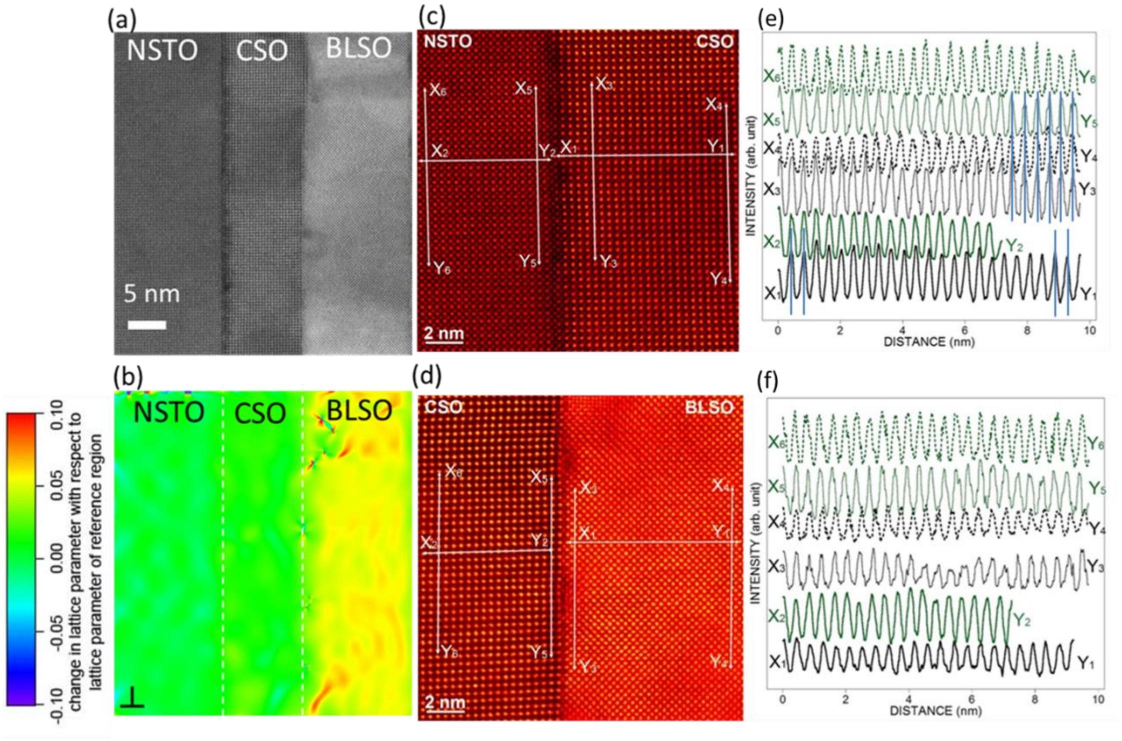

Supplementary Fig. S2. HAADF STEM images, GPA map along the growth direction, and line profiles of atomic spacing across the full BLSO/CSO/NSTO heterostructure. (a) Grey scale HAADF STEM image and (b) geometric phase analysis (GPA) map along the growth direction (denoted by ⊥) across the full BLSO/CSO/NSTO structure. For the GPA map of (b) the reference region is the NSTO region. Owing to the lower resolution of the STEM image required to image the full structure, the GPA map (as well as all GPA maps in the supplementary material) exhibits lower signal to noise ratio than the GPA maps acquired at higher resolution shown in Figs. 1 and 2 of the main text. (c) presents an enlarged color HAADF STEM image showing the NSTO and CSO lattice across the width of the CSO film while (d) shows the corresponding enlarged HAADF STEM image of the CSO film and top BLSO layer. (e) and (f) display the line profiles of the lattice spacing along the growth (lines X_1Y_1 and X_2Y_2) and in-plane directions (lines X_3Y_3, X_4Y_4, X_5Y_5, and X_6Y_6) for the structures of panels (c) and (d), respectively. The blue lines in (e) are a guide to the eye. The line profiles demonstrate a CSO lattice spacing in-plane that is coherent with the NSTO substrate in-plane lattice spacing (compare line profiles X_3Y_3, X_4Y_4 and X_5Y_5 in (e) which exhibit peaks occurring at the same location across the full 10 nm length of the in-plane line profile). The CSO film also exhibits a constant lattice spacing across the film along the

growth direction (compare the distance between peaks at the end points of line X_1Y_1). The constant lattice spacing across the 10 nm line profile is consistent with the small error in the in-plane lattice parameter of ± 0.02 Å derived from the FWHM along the in-plane direction of the 103 RSM CSO peak FWHM. The GPA maps and line profiles demonstrate the strain does not occur only near the interface(s) but exists throughout the CSO film volume.

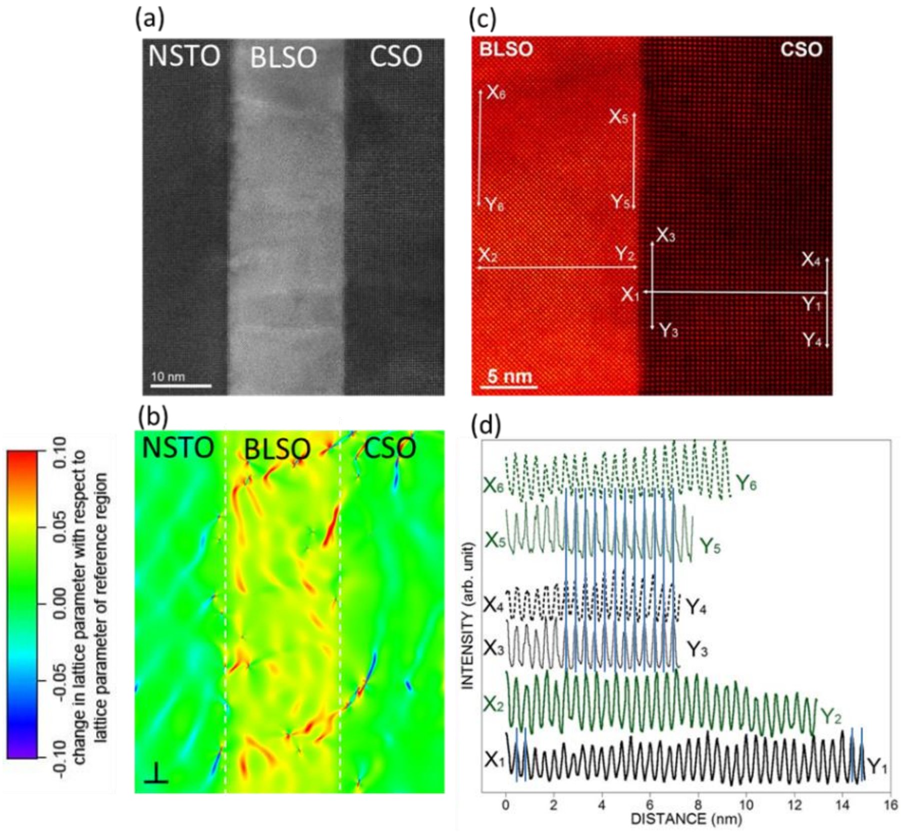

Supplementary Fig. S3. HAADF STEM images, GPA map along the growth direction, and line profiles of atomic spacing across the full CSO/BLSO/NSTO heterostructure. Gray scale (a) HAADF STEM image and (b) GPA map along the growth direction (denoted by ⊥) across the full CSO/BLSO/NSTO structure. For the GPA map of (b) the reference region is the NSTO region. (c) presents an enlarged color HAADF STEM image showing the BLSO and CSO lattice across their respective film thicknesses while (d) displays line profiles of the lattice spacing along the growth (lines X_1Y_1 and X_2Y_2) and in-plane directions (lines X_3Y_3, X_4Y_4, X_5Y_5, and X_6Y_6) as illustrated in (c). As revealed by comparing in-plane line profiles X_3Y_3 or X_4Y_4 in the CSO film to line profiles X_5Y_5 or X_6Y_6 in the BLSO film the CSO film is not coherently

strained to the BLSO film which is consistent with the RSM of figure 1(j) of the main text. However, constant lattice spacing is observed in the CSO along a line along the growth direction (compare the distance between peaks at the end points of line X_1Y_1). In contrast to the coherently strained CSO film on NSTO (Supplementary Fig. S2), the in-plane line profiles indicate a larger mosaic spread. Note the in-plane line profiles X_3Y_3 and X_4Y_4 in (d) are in phase up to ~5 nm length in-plane after which the line profiles exhibit a phase difference corresponding to a difference in lattice constant of ~0.1 Å. This is consistent with the larger error (± 0.13 Å) in the in-plane lattice parameters derived from the FWHM along the in-plane direction of the (103) RSM CSO peak presented in Table 1 in the main text.

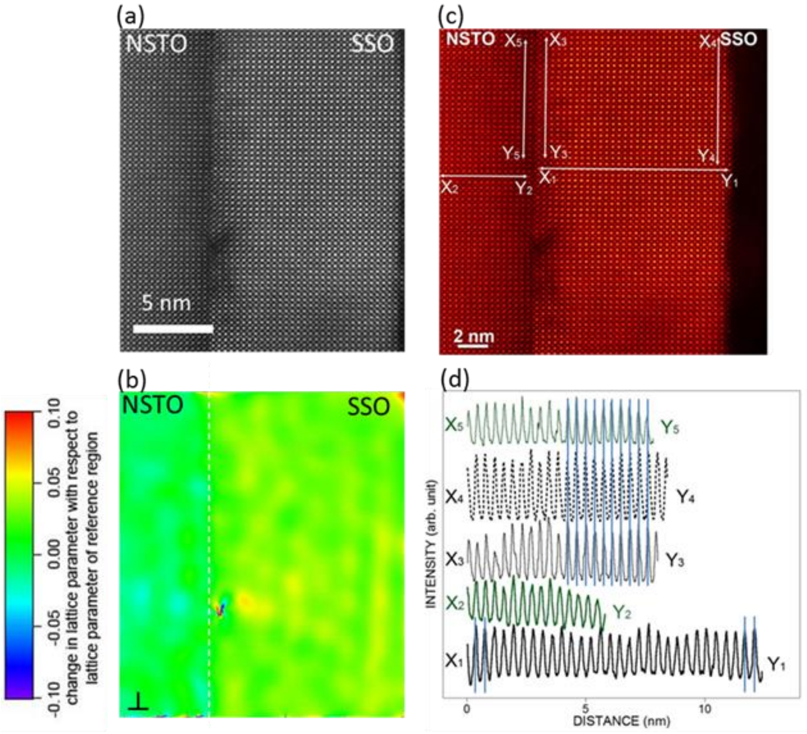

Supplementary Fig. S4. HAADF STEM images, GPA map along the growth direction, and line profiles of atomic spacing across the full SSO/NSTO heterostructure. (a) Gray scale HAADF STEM image and (b) GPA map along the growth direction (denoted by ⊥) across the full SSO/NSTO structure. For the GPA map of (b) the reference region is the NSTO region. (c) presents a color HAADF STEM image showing the locations taken for the line profiles of the lattice spacing along the growth (lines X_1Y_1 and X_2Y_2) and in-plane directions (lines X_3Y_3, X_4Y_4, and X_5Y_5) while (d) presents the line profiles

along the lines illustrated in (c). While a misfit dislocation is observable in the HAADF STEM image and GPA map, both the GPA maps and line profiles indicate that, away from the lattice defect, the SSO is coherently strained to the NSTO substrate (compare line profiles lines X_3Y_3, X_4Y_4, and X_5Y_5) and has uniform lattice spacing along the growth direction (compare the distance between peaks at the end points of line X_1Y_1). The in-plane line profiles also exhibit uniform spacing between peaks over ~ 8 nm length in-plane. The line profiles and STEM/GPA data are consistent with the RSM of Fig. 2(a) in the main text that shows the SSO (103) peak position coherent with the substrate peak position but also exhibits a larger broadening of the RSM peak (± 0.05 Å) as compared to the CSO film on NSTO (± 0.02 Å), which has smaller misfit strain and misfit dislocations were not observed. The data indicates the strain is not only at the interface but exists throughout the SSO film volume.

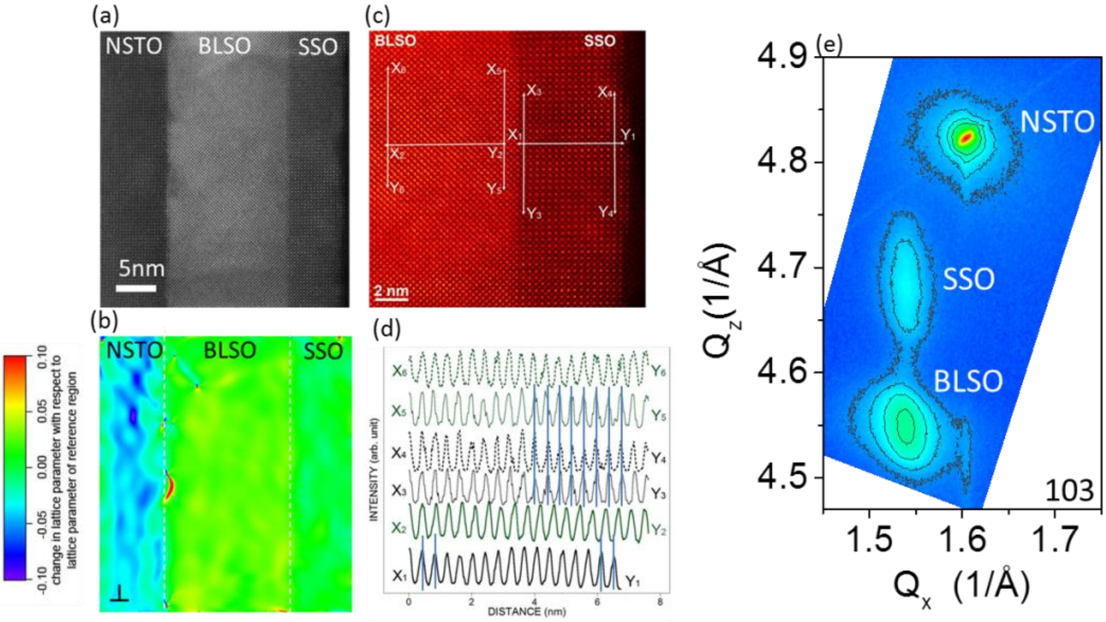

Supplementary Fig. S5. HAADF STEM images, GPA map along the growth direction, and line profiles of atomic spacing across the full SSO/BLSO/NSTO heterostructure. (a) Gray scale HAADF STEM image, (b) geometric phase analysis (GPA) map along the growth direction (denoted by ⊥), (c) color HAADF STEM image showing the locations taken for the line profiles of the lattice spacing along the growth (lines X_1Y_1 and X_2Y_2) and in-plane directions (lines X_3Y_3, X_4Y_4, X_5Y_5, and

X_6Y_6), (d) line profiles across a full SSO(7nm)/BLSO/NSTO structure, and (e) RSM for the same structure. For the GPA map of (b) the reference region is the SSO region. Consistent with the RSM, the in-plane line profiles show $a_{SSO} \approx a_{BLSO}$ for the SSO and BLSO in-plane lattice parameters, respectively. A constant lattice spacing is also observed along a line along the growth direction across the film (compare the distance between peaks at the end points of line X_1Y_1). The GPA map and line profiles show the strain exists throughout the SSO film volume. The in-plane and out-of-plane lattice parameters are a_{SSO} = 4.078 ± 0.08 Å and c_{SSO} = 4.027 ± 0.02 Å, respectively, which are, within error, the same as those derived from the RSM of the 11 nm SSO film grown on BLSO/NSTO shown in Fig. 2(f) of the main text which has lattice parameters a_{SSO} = 4.075 ± 0.08 Å and c_{SSO} = 4.021 ± 0.01 Å.

.

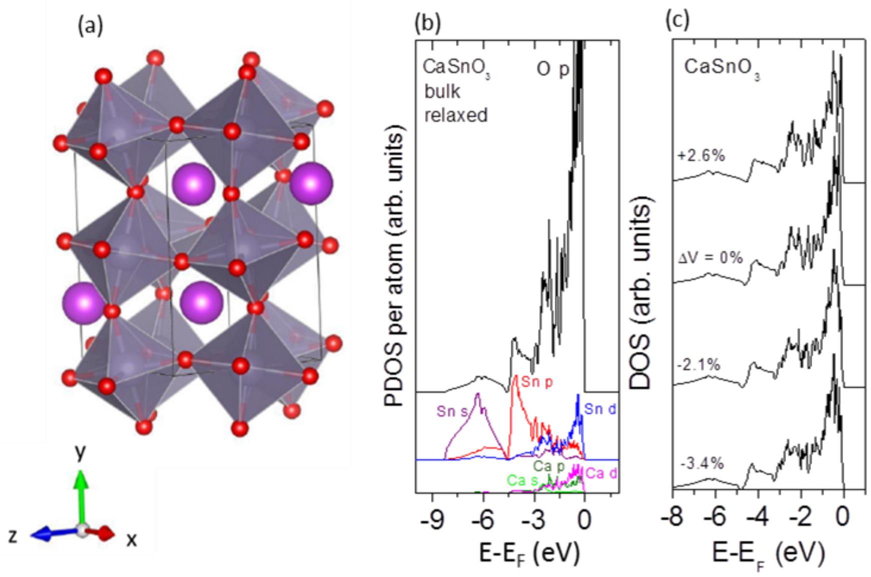

Supplementary Fig. S6. PDOS per atom for $CaSnO_3$ with *Pbnm* orthorhombic symmetry and DOS for various volumetric strain of bulk orthorhombic $CaSnO_3$. (a) Pictorial representation of $CaSnO_3$ with *Pbnm* orthorhombic symmetry. Red spheres represent oxygen with each oxygen octahedron enclosing a Sn atom while purple spheres represent Ca. (b) Projected density of states (PDOS) per atom for $CaSnO_3$ with *Pbnm* orthorhombic symmetry. (c) DOS for various volumetric strain of bulk orthorhombic $CaSnO_3$ achieved by compressing or expanding the unit cell in two dimensions (the XZ plane as shown in (a)).

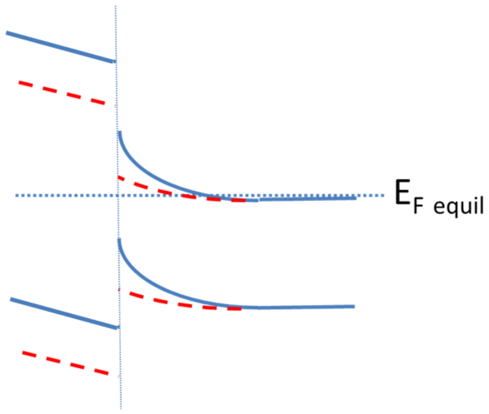

Supplementary Fig. S7. Schematic band diagram accounting for the apparent decrease in the measured VBO for CSO and SSO stepwise grown on NSTO.

In equilibrium (blue curves) a depletion layer is formed in the NSTO during interface formation with CSO or SSO. Owing to the depletion charge in the NSTO and Gauss's law, an internal field exists in the CSO or SSO film, causing the CSO or SSO bands to slope upwards. Under a photon flux (dashed red curves) the film (left side of the interface indicated by the vertical blue line) and substrate (right side of the interface) energy levels (conduction band, valence band (VB), and core levels (CLs)) are shifted downwards (the VB and CLs are shifted towards higher binding energy (HBE) in the photoemission spectra) due to a PV resulting from the separation of photoexcited electron-hole pairs in the depletion region. In the CSO or SSO film, an internal field, supported by line shape broadening (see Supplementary Fig. S8 online), results in an upward sloped potential gradient (from substrate to film surface) partially compensating the HBE shift of the CSO or SSO CLs at the interface with increasing depth into the film, resulting in an apparent decrease in the measured VBO determined by monitoring the thickness evolution of the CLs.

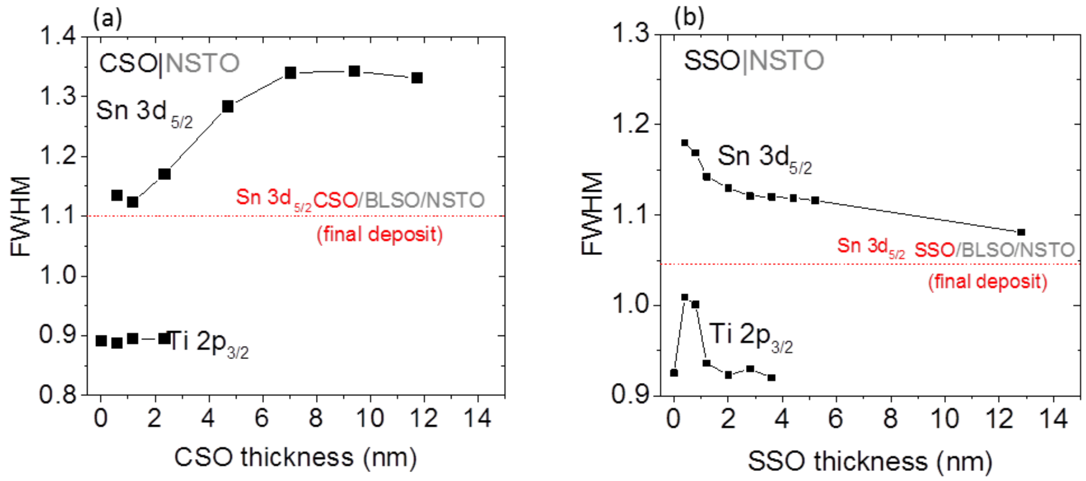

Supplementary Fig. S8. Thickness evolution of the full width half maximums for the Sn $3d_{5/2}$ and Ti $2p_{3/2}$ core levels. Thickness evolution of the full width half maximums (FWHM) for the Sn $3d_{5/2}$ and Ti $2p_{3/2}$ CLs during stepwise growth of (a) CSO and (b) SSO on NSTO. Also shown on the plots are the FWHM for the Sn $3d_{5/2}$ CL of the final deposit of CSO and SSO on BLSO/NSTO as shown by the dotted red line in (a) and (b), respectively. The FWHMs evolve with thickness in a manner that approximately (inversely) correlates with the CL binding energy evolutions as revealed by comparing (a) and (b) to Figs. 6(f) and 6(c), respectively in the main text which present the CL BE evolution for CSO and SSO stepwise deposited on NSTO. Note in particular that for SSO stepwise deposited on NSTO the FWHM of the Ti $2p_{3/2}$ CL emission for the NSTO substrate increases and subsequently decreases resulting in a peak-like feature while over the same SSO deposit thickness range the Ti $2p_{3/2}$ CL emission shown in Fig.6 (c) shifts to LBE then HBE resulting in a dip-like feature in Fig. 6(c). For both CSO and SSO stepwise deposited on NSTO, the FWHM are wider than the FWHM of the final deposit CSO and SSO films on BLSO/NSTO. These trends are consistent with internal field induced changes in the FWHM where shifts of CL emissions to LBE are accompanied by larger FWHMs owing to larger internal fields in the NSTO n-type depletion layers or, for the films, upward sloped potential gradients as shown in Fig. S7. The larger FWHM results from a superposition of CL emissions with local BEs that vary continuously with depth owing to the potential gradients.

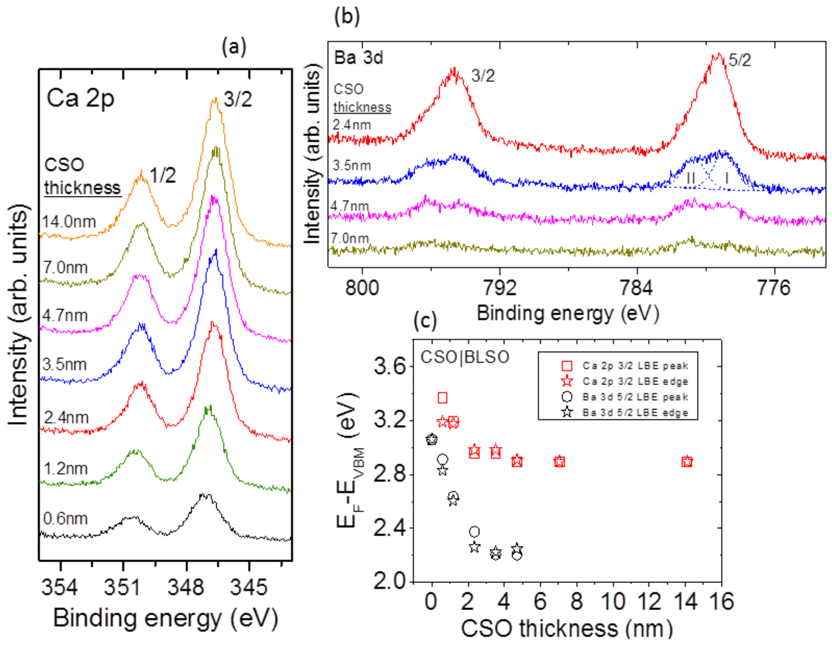

Supplementary Fig. S9. Thickness evolution of the Ca 2p and Ba 3d core levels for stepwise growth of CSO on BLSO/NSTO. Thickness evolution of the (a) Ca 2p and (b) Ba 3d core levels for stepwise growth of CSO on BLSO/NSTO. The Ca 2p CL line shape is characterized by a single spin orbit split (SOS) doublet while the Ba 3d core level line shape is asymmetric and can be synthetically resolved into two SOS doublets with FWHM of 1.4 eV and separated by 1.7 eV. Owing to the thickness dependence, the HBE doublet is associated with Ba at the CSO|BLSO interface while the LBE component is associated with Ba in the BLSO film bulk. Such HBE components of Ba 3d CLs are often observed for surface Ba atoms (Baniecki, J. D., Ishii, M., Shioga, T., Kurihara, K. & Miyahara, S. Surface core-level shifts of strontium observed in photoemission of barium strontium titanate thin films. Applied Physics Letters 89, 162908-1~3 (2006)). Either synthetically resolving the two SOS doublets and using the LBE component to monitor CL evolution (denoted “LBE peak” in (c)) or extrapolating the leading edge of the LBE component to the background (denoted “LBE edge” in (c)) yielded the same thickness evolution of core level binding energies. The data in the Fig. 6(d) of the main text presents the “LBE peak” data of (c).

Supplementary Table S1. Lattice parameters for all thin film layers of the fabricated heterostructures. In-plane, a, and out-of-plane, c, lattice parameters for all thin film layers of the fabricated heterostructures. The thickness of each layer is also shown to the right of the layer. The errors are estimated from the RSM full width half maximum (103) peak along the <100> and <001> directions of k-space, respectively. For the multilayer structures the top-most entry is the top thin film layer and the bottom most-entry the bottom thin film layer in the heterostructure stack.

Structure<br>(on NSTO)	In-plane lattice parameter<br>a (Å)	Out-of-plane lattice parameter<br>c (Å)
BLSO (20 nm)	4.112 ± 0.08	4.129 ± 0.04
SSO (11 nm)	3.919 ± 0.05	4.136 ± 0.02
SSO (11 nm)<br>BLSO (17 nm)	4.075 ± 0.08<br>4.084 ± 0.08	4.021 ± 0.01<br>4.144 ± 0.02
La-STO (12 nm)<br>SSO (11 nm)<br>BLSO (17 nm)	3.985 ± 0.02<br>4.069 ± 0.16<br>4.122 ± 0.08	3.902 ± 0.02<br>4.022 ± 0.02<br>4.135 ± 0.02
CSO (12 nm)	3.905 ± 0.02	3.981 ± 0.05
BLSO (15 nm)<br>CSO (12 nm)	4.088 ± 0.11<br>3.918 ± 0.02	4.149 ± 0.05<br>3.977 ± 0.13
CSO (17 nm)<br>BLSO (20 nm)	3.979 ± 0.14<br>4.102 ± 0.11	3.922 ± 0.06<br>4.131 ± 0.04

Supplementary Table S2. DFT calculation parameters. DFT calculated cell parameters and cell volume V_{cell} for the relaxed orthorhombic $SrSnO_3$ (SSO) and $CaSnO_3$ (CSO) structures and structures that have been compressed or expanded in 2 dimensions (2D) along the short axes of the cell (along a_x, a_z) to mimic epitaxial strain. Also shown are the percent volumetric strain ΔV_{pc} % with respect to the relaxed structures, the optical gap $E_{G\ opt}$, the fundamental gap $E_{G\ fund}$, and the energy separation ΔVBM between the VB maximums for the optical and fundamental band gaps.

Material/ structure	a_x (Å)	a_y (Å)	a_z (Å)	V_{cell}x10^{-3} (Bohr^3)	ΔV_{pc} %	$E_{G\ opt}$ (eV)	$E_{G\ fund}$ (eV)	ΔVBM (eV)
SSO relaxed	5.679	7.990	5.648	1.72936	0	2.33	2.24	0.09
SSO 2D compressed	5.455	8.275	5.455	1.66204	-3.9	2.75	2.74	0.01
SSO 2D expanded	5.720	7.931	5.720	1.75106	1.25	2.19	2.12	0.07
CSO relaxed	5.645	7.820	5.468	1.62901	0	2.88	2.75	0.13
CSO 2D compressed	5.455	7.937	5.455	1.59410	-2.14	3.05	2.96	0.09
CSO 2D expanded	5.665	7.718	5.665	1.67157	2.6	2.53	2.51	0.02
